# Do children unintentionally report maltreatment? Comparison of disclosures of neglect versus sexual abuse

**DOI:** 10.1016/j.chiabu.2022.105824

**Published:** 2022-08-12

**Authors:** Jennifer Lavoie, Shanna Williams, Thomas D. Lyon, Jodi A. Quas

**Affiliations:** a University of Edinburgh, Moray House School of Education & Sport Holyrood Rd, Edinburgh EH8 8AQ, United Kingdom; b McGill University, Department of Educational and Counselling Psychology Education Building, 3700 McTavish St, Montreal, Quebec H3A 1Y2, Canada; c University of Southern California, Gould School of Law, 699 Exposition Boulevard, Los Angeles, CA 90089-0071, United States of America; d University of California, Irvine, Department of Psychological Science, 4328 Social and Behavioral Sciences Gateway, Irvine, CA 92697, United States of America

**Keywords:** Child maltreatment, Investigative interviewing, Disclosure, Awareness

## Abstract

**Background and aims:**

Children’s initial reports often play a key role in the identification of maltreatment, and a sizeable amount of scientific research has examined how children disclose sexual and physical abuse. Although neglect constitutes a large proportion of maltreatment experiences, relatively little attention has been directed toward understanding whether and how children disclose neglect. The overarching aim of the present study was to document this process by comparing disclosure patterns in cases of neglect to those in cases of sexual abuse.

**Method:**

Redacted jurisdiction reports (*N* = 136) of substantiated dependency cases of neglect (*n* = 71) and sexual abuse (*n* = 65) in 4- to 17-year-olds were coded for why maltreatment was suspected, and for children’s perceived awareness and disclosure of the maltreatment.

**Results:**

Neglect was most often initially suspected via contact with emergency services (e.g., police, emergency medical services), whereas sexual abuse was most often initially suspected as a result of children’s statements. Children evidenced greater perceived awareness of sexual abuse than neglect and were more likely to disclose the former in their first investigative interview. Perceived awareness was further associated with a higher likelihood of children’s statements initiating discovery of maltreatment and disclosing in the first investigative interview.

**Conclusions:**

Children may benefit from greater knowledge about their needs for safety, supervision, and provision in the home, which could increase the likelihood they would disclose neglect. Such, in turn, could lead to earlier interventions for children and families.

## 1. Introduction

Given that the vast majority of maltreatment occurs out of the public’s eye, children’s initial reports are often catalysts for identification of victimization (Heger et al., 2002; Lyon et al., 2012; Rush et al., 2017). Indeed, a large body of research has been devoted to understanding how children disclose, most often sexual abuse but also physical abuse, with the broader goal of identifying ways of increasing those disclosures without compromising accuracy (e.g., Brubacher et al., 2013; Malloy et al., 2015; McElvaney et al., 2020; Okur et al., 2020; Rush et al., 2014). Yet, another form of maltreatment, neglect, also tends to occur in private and typically lacks overt or clear signs of its occurrence. How children disclose neglect and whether their disclosures play key roles in its identification are largely unknown; surprising omissions given that neglect comprises the most common form of maltreatment (Gonzalez et al., 2021). Thus, it is particularly important to understand how children disclose neglect. The current study was designed to provide this understanding, focusing on how neglect is discovered, whether children are aware of neglect, and how that awareness relates to their disclosures. We specifically compared discovery, awareness, and disclosures between cases involving neglect and sexual abuse substantiated by social services to determine how, and potentially why, disclosure varies between the two types of maltreatment.

Extant literature suggests that the ways in which neglect versus sexual abuse are discovered vary. With sexual abuse, the most common way it is discovered is via children’s own disclosures, which often occur following lengthy delays (Alaggia et al., 2019; Goodman et al., 1992; London et al., 2005). Children’s initial reports may be made to peers, parents, or other adults, who either call authorities or tell someone who does (Heger et al., 2002; Lyon et al., 2012; Priebe & Svedin, 2008). Many such disclosures appear tentative or unintentional. Older children, for instance, may be testing the waters to gauge others’ reactions to their reports; younger children may not fully understand their experiences but may nonetheless reveal information during conversations with others that raise suspicions or concerns worthy of reporting (Anderson, 2016; Priebe & Svedin, 2008). Only a paucity of research has considered such types of disclosures (i.e., those that may be tentative or unintentional). It is possible, though, that children’s perceived awareness of the types of behaviors that constitute maltreatment may factor into whether they intentionally report or not, as was examined in this study.

Neglect, in contrast to sexual abuse, is most often identified when a third party, such as members of a child’s extended family, teacher or neighbor, report concerns based on their interactions with or observations of the child and family (U.S. Department of Health et al., 2019). The report then initiates a formal investigation. Yet, third party individuals’ reporting tendencies are unlikely to adequately capture some or perhaps even a majority of cases of neglect. For one, although such individuals may be able to identify obvious omissions of care, security, or shelter (Dubowitz et al., 1998; Portwood, 1998; Rose & Selwyn, 2000), they may have difficulty distinguishing factors related to poverty from neglect (e.g., unstable housing situation but supportive caregiving; Dickerson et al., 2020), leading to over- or under-reporting of children who suffered actual neglect. Second and potentially related, suspecting and reporting neglect are not identical. Some individuals who have concerns about a child may decide not to contact the authorities, for instance, because they do not believe that the situation is serious enough to warrant reporting (McDaniel, 2006; Portwood, 1998) or because they do not believe that the authorities can actually help, for example due to mistrust in social services (Griffin, 2008; Meysen & Kelly, 2017). And third, some forms of neglect, such as failure to protect, are not linked to lack of care or shelter and are unlikely to have overtly visible signs that individuals could see and report.

A second way that neglect can be identified is via children’s own reports. When questioned in investigative interviews about maltreatment of themselves or others (e.g., a sibling), for example, children may provide additional information suggestive of neglect that warrants further investigation. Alternatively, children may inadvertently report suspicious details about their home life to others that prompt follow up or reporting by a third party. In both situations, children’s disclosures may reflect their knowledge of harm or risk or may be done unintentionally with little overt awareness that their experiences constitute a form of neglect.

Regardless of how either sexual abuse or neglect is initially suspected, the purpose of the investigative interview is to determine whether maltreatment has actually occurred, and if so, the perpetrator(s)’ identity and what the extent of ongoing or future harm is. For such a determination, children’s disclosures may be crucial (Finkelhor & Wolak, 2003). Some children, however, may not understand or recognize their experiences as maltreatment, which could impede their disclosure. In their minds, there is nothing to disclose. Evidence that this may be occurring has emerged in studies of children immersed in the dependency system. Block et al. (2010), for instance, reported that some of the 7–10 year-olds attending dependency court hearings following removal from the home due to maltreatment did not seem to adequately understand why they were in court, which could reflect a lack of awareness that they had been maltreated (or what maltreatment is). Moreover, in investigations of foster children’s perceptions of their removal from home, many report thinking that they were in trouble rather than they were at risk of serious harm (Folman, 1998).

Of note, a lack of awareness about maltreatment may well be more common among children exposed to neglect than sexual abuse. Children are often taught from a fairly early age about body parts, boundaries, and inappropriate touching (Kemshall & Moulden, 2017). As a result, if experiences relevant to such touching do occur, children would presumably be more likely to realize that such experiences are wrong than forms of maltreatment about which such teaching is minimal or non-existent. Yet, at the same time, cases of sexual abuse presented by the media, such as that against hundreds of young gymnasts committed by a gymnastics doctor in the USA (e.g., Hauser & Astor, 2018) do suggest that knowledge of inappropriate touching may not translate as easily as might be expected, though the fact that this abuse was under the guise of medical care does raise the question of whether knowledge of abuse might have been more clear under other circumstances.

Nonetheless, neglect, unlike sexual abuse, may not involve discrete events or experiences that children could pinpoint and recount, as they do other autobiographical events. Explicit questions raise concerns about suggestibility (see Hritz et al., 2015 for a review; Quas et al., 2007; Saywitz et al., 2018), leading well-trained investigators to avoid posing such questions. Insofar as children are unaware of their own maltreatment, they would be unlikely to disclose, not because they are intentionally hiding what happened, which occurs at times with sexual abuse, but instead because they do not have anything to report. With age, children’s recognition that neglectful experiences constitute maltreatment could increase due to greater exposure to non-familial contexts (e.g., friends’ homes, school, extra-curricular activities, religious activities), although recognition does not necessarily translate into disclosure, as intentional disclosures may drop with age as children’s understanding of the consequences of reporting maltreatment likely increase (Azzopardi et al., 2019).

In summary, children’s own reports most often prompt investigations of suspected sexual abuse. These reports are often intentional, especially among older children who seem to have some understanding of the harmful and wrongful nature of their experiences and of the consequences of disclosing (e.g., Priebe & Svedin, 2008). These reports, moreover, are also predictive of later formal disclosures to the authorities (Grandgenett et al., 2019), although some children, especially those who are younger, may initially report sexual abuse, for instance, to a trusted adult, but then recant their claim when questioned formally by an investigative interviewer (Azzopardi et al., 2019; Malloy et al., 2007). In contrast, investigations of neglect are prompted by concerned others who report their suspicions or possibly by children’s unintentional reports, and children may not be aware that their experiences constitute neglect. Initial unintentional reports may then be followed by nondisclosures when formally questioned by the authorities. With age, given that children’s awareness of their own neglect and of the consequences of disclosing both increase, intentional reports may also be followed by nondisclosure of neglect in formal interviews, a pattern sometimes observed with sexual abuse.

### 1.1. Current study

The overarching goal of this study was to provide much-needed insight into how children disclose neglect. To do so, we compared discovery, awareness, and investigative interview disclosure patterns between children who experienced substantiated neglect and substantiated sexual abuse. Based on extant literature, we first expected sexual abuse, more so than neglect, to be initially suspected as a result of children’s own statements (Heger et al., 2002; Lyon et al., 2012; Rush et al., 2017). Second, we anticipated that, across age, children exposed to sexual abuse would be more likely than children exposed to neglect to be aware of the harmful (i.e., abusive) nature of their experiences. Third, we predicted that this awareness would further predict a greater likelihood of disclosure in the first investigative interview. Finally, we hypothesized that, among both types of maltreatment, developmental differences would emerge, such that increasing age would be related to greater awareness of the maltreatment.

## 2. Method

### 2.1. Participants

Data were derived from 136 dependency cases involving substantiated neglect or sexual abuse for which jurisdiction reports (i.e., detailed reports on the investigation and its conclusions provided to the courts) were available. None of the children in these cases had been involved in a documented prior child protection investigation to avoid past involvement affecting perceived awareness of parent/guardian behavior, and all maltreatment was deemed sufficiently serious to warrant children’s removal from the custody of their parents. The alleged perpetrator in all cases was a parent or family member. Finally, only one child per case was eligible.

In addition, for the neglect sample, children only experienced substantiated neglect (i.e., no physical or sexual abuse or domestic violence) to avoid conflation of children’s perceived awareness of types of maltreatment that may be more overt. For the sexual abuse sample, all children had experienced substantiated sexual abuse, but many had experienced other forms of maltreatment as well, including physical abuse (23 cases), neglect-failure to provide (24 cases) and neglect-failure to protect (e.g., witnessed domestic violence; 19 cases).

The final sample included 71 cases involving only neglect, and 65 involving sexual abuse. In the neglect sample, children’s ages ranged from 4 to 17 years (*M* = 11.4 years, *SD* = 3.26 years), and 61 % were female. In the sexual abuse sample, children’s ages sample also ranged from 4 to 17 years (*M* = 11.1 years, *SD* = 2.58 years), and 91 % were female. *t*-Test comparisons indicated that the two samples were similar in age *t*(131.40) = 0.63, *p* = .53, but there were more females than males in the sexual abuse sample, *t*(115.44) = −4.40, *p* < .001.

### 2.2. Measures and procedures

Following approval from the Presiding Judge of Juvenile court to access and anonymize children’s information, and university ethical approval to utilize the archived anonymized materials, case details were coded for children living in out of home care and had participated in other ongoing studies of maltreatment and legal involvement effects on children (e.g., Dickerson et al., 2018; Malloy et al., 2016; Quas et al., 2009; Wandrey et al., 2012). From these, case files were reviewed to identify children within the target age range who met the study criteria.

The following information was then extracted from each eligible case file (reliability established on 20 % of case files with 95 % agreement, disagreements resolved by mutual approval): (1) all maltreatment allegations and outcomes; (2) the child’s home and family life (e.g., living conditions, such as whether the home was safe, clean, and included food and water; parental supervision such as whether child was left alone for long periods of time); (3) the nature of the events contributing to the suspicion and eventual discovery of maltreatment (e.g., call from neighbor, child tells friend, teacher suspicion); (4) police or emergency service responses (e.g., medical evaluation); and (5) details regarding any behaviors or statements the child provided relevant to the maltreatment, with a timeline indicating when these were provided (from the first suspicions to the first investigative interview). From this information, the following reliably coded variables were of interest.

#### 2.2.1. Demographic and case context variables

The child’s age and gender were documented, as was each type of substantiated maltreatment. Types distinguished neglect, sexual abuse, physical abuse, and other (e.g., emotional abuse), from which the two study groups, neglect versus sexual abuse, were created. We also recorded whether the child had been exposed to domestic violence. For the neglect sample, cases that contained exposure to domestic violence were excluded.

#### 2.2.2. Initial discovery of maltreatment

**Fig. 1.**
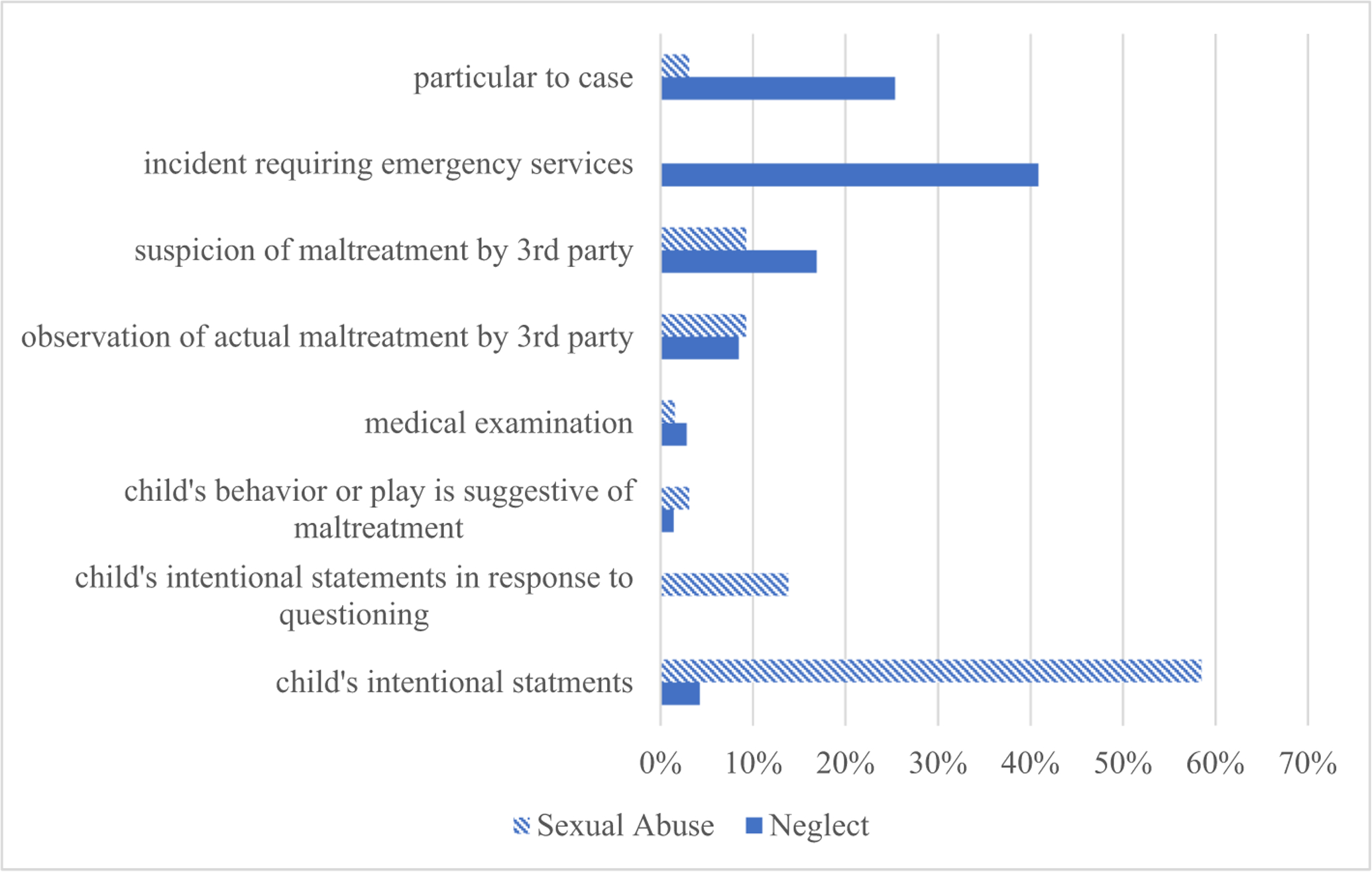
Ways in which maltreatment was initially discovered.

From all of the reasons that maltreatment had been suspected across the cases (e.g., child told a friend about the maltreatment, law enforcement showed up at a home because a gun shot was heard), a dichotomous variable, initial suspicion, was created to reflect whether the child’s report initiated (1) or did not initiate (0) the maltreatment investigation (Fig. 1).

#### 2.2.3. Perceived awareness

A dichotomous code was created to indicate children’s reported understanding or awareness that their experiences constituted maltreatment (neglect or sexual abuse). Unawareness, coded as 0, was characterized by a naiviete in the child’s reported behavior or statements about the maltreatment. Examples include a child expressing confusion about why law enforcement or social workers were asking about the child’s circumstances (e.g., living situation, relationship or experiences with perpetrator) or the child describing her experiences or the situation as normal. Awareness, coded as 1, was characterized by a child recognizing a problem, or something undesirable about their experiences. Sample indicators included a child expressing clear disapproval of a parent (“bad father”) or a parent’s actions, or describing living circumstances as wrong or harmful. Any indicator of awareness, even if other indicators suggested unawareness, was prioritized in coding (i.e., we coded as aware if there were any indicators of awareness). Of importance, awareness was coded separate from investigative interview disclosure (described next), given that a child could disclose an experience or situation unintentionally, without being fully cognizant of its designation.

#### 2.2.4. Disclosure in first investigative interview

We identified the first investigative interview documented in children’s files and coded children’s statements into one of four mutually exclusive categories: (1) no disclosure (e.g., the child’s responses revealed nothing of concern), (2) explicit denial (e.g., the child verbally denied all wrongdoing), (3) partial disclosure (e.g., the child provided information suggestive of harm or risk but did not provided a clear report), and (4) explicit disclosure (e.g., the child provided detailed information confirming that maltreatment had occurred).

## 3. Results

Preliminary analyses assessed the relations between the three main outcomes of interest (initial discovery, awareness, and disclosure), as well as age. Results are reported in Table 1. No direct age differences emerged across the outcomes of interest, but given that age at times is related to patterns of disclosure more generally (e.g., Azzopardi et al., 2019; Leach et al., 2017), age was included in our statistical models. Because so few boys (*n* = 6) were included in the sexual abuse sample, we were unable to conduct analyses with gender and maltreatment in the same model. Nonetheless, in the neglect sample, when we tested for gender differences in discovery, awareness, and disclosure (*n* = 28 boys), no gender differences emerged. Thus, while gender may be important to consider overall with regard to children’s reporting and disclosure of maltreatment, especially sexual abuse, it is unlikely to be the primary source of evident differences in the current sample between reporting, awareness, and disclosure patterns.

### 3.1. Discovery of maltreatment

As predicted, neglect was discovered differently than was sexual abuse. Neglect was most commonly initially suspected not because of the child, but because of some other indicator. This included (1) general emergency incidents (41 %, *n* = 29; e.g., police called to the scene due to a concern of a neighbor or passer-by who saw a child in a perceived unsafe situation, or situations requiring emergency medical services), (2) circumstances particular to the case (25 %, *n* = 18; e.g., sibling born with illegal substances in system) or (3) suspicion by a third party, for example a neighbor or teacher (17 %, *n* = 12; e.g., a neighbor noticed very young children regularly playing in the streets without supervision). In fact, only 4 % of the neglect investigations were launched as a result of the child’s intentional statements (*n* = 3; e.g., a child ran away from the parent’s home after the parent offered the child drugs and the child told a family friend). A descriptive breakdown of the ways in which maltreatment was initially suspected is presented in Fig. 1.

**Fig. 2.**
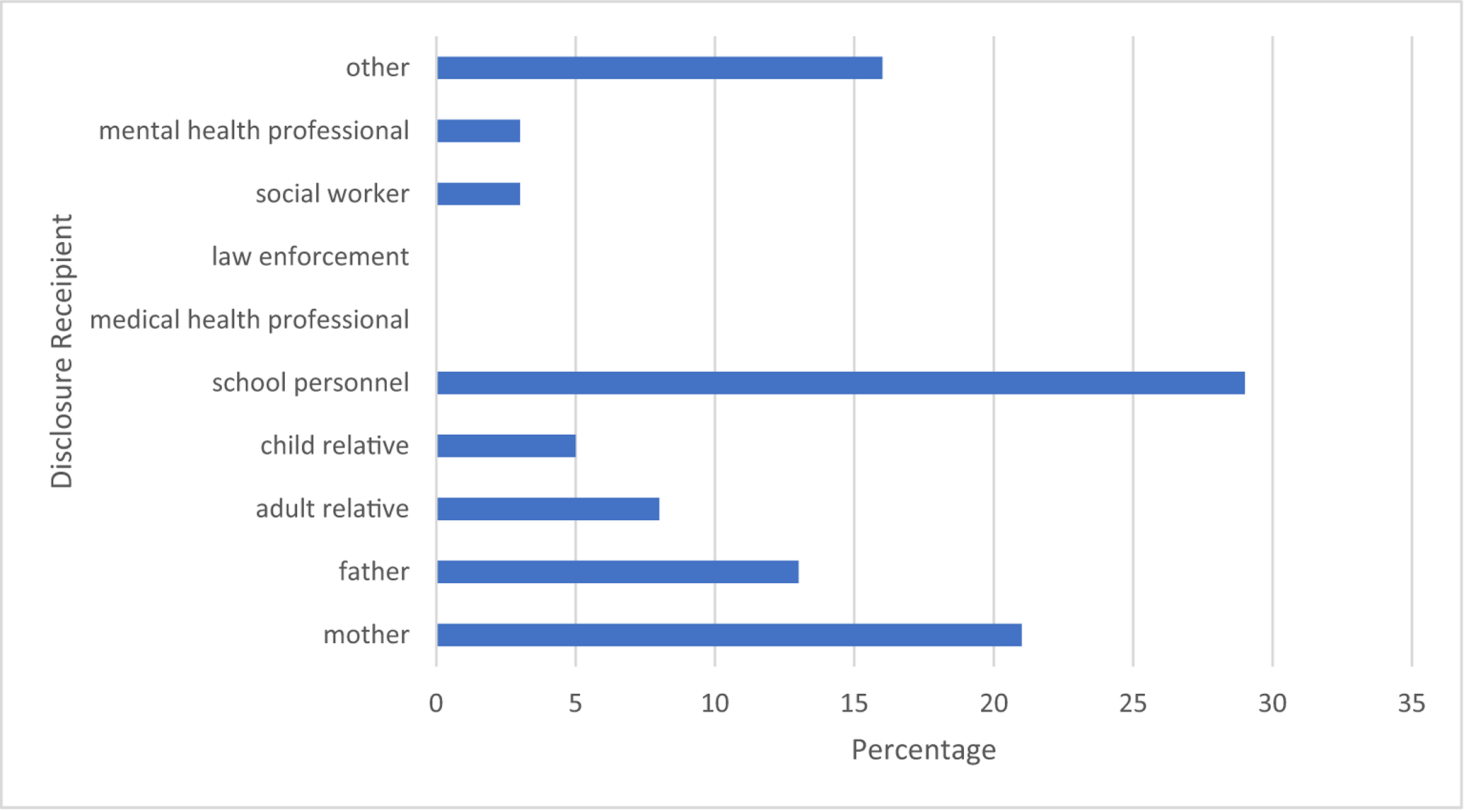
Breakdown of disclosure recipients of children’s initial reports (discovery) in the sample of children who had experienced sexual abuse.

In contrast, sexual abuse was significantly more likely to be discovered as result of the child’s own report. In the majority of cases, 58 % (*n* = 38), the child initiated a report to someone who then reported the abuse to the authorities (specific percentages of who these initial disclosures were made to are presented in Fig. 2); The second most common way sexual abuse was discovered involved suspicions by another person (e.g., mother, sibling) who then solicited abuse details from the child (14 %, *n* = 9). Finally, smaller percentages of cases were initially identified via observation of abuse or suspicious of abuse by a third party (9 %, *n* = 6, observed, 9 %, *n* = 6, suspicion), without any direct questioning of the child.

### 3.2. Predicting awareness, discovery, and disclosure

The main goals were to understand the links between how the maltreatment was discovered and children’s awareness, and then how these independently and jointly related to children’s formal disclosure.

First, we considered whether age and type of maltreatment predicted perceived awareness (dichotomous) via a logistic regression. The model was significant, *χ*^*2*^(2, *N* = 134) = 34.06, *p* < .001, Nagelkerke’s *R*^*2*^ = 0.30 (Table 2), with maltreatment type emerging as a significant predictor, *b* = 1.09, *p* < .001, OR = 2.97. Children who had experienced neglect were nearly three times less likely than children who had experienced sexual abuse to demonstrate perceived awareness of their experiences as maltreatment.

Second, we sought to determine whether children’s awareness was associated with initial discovery of maltreatment. We conducted a logistic regression with age, type of maltreatment, and perceived awareness as predictors and the dichotomous variable of whether the child initiated a first report (yes/no) as the outcome. The overall model was significant, *χ*^*2*^(3, *N* = 133) = 88.52, *p* < .001, Nagelkerke’s *R*^*2*^ = 0.66. Both type of maltreatment, *b* = −2.06, *p* < .001, OR = 0.13, and awareness, *b* = −1.45, *p* = .017, OR = 0.23, significantly predicted the child initiating a first report (Table 2). Children who had been neglected were 87 % less likely to initiate a first report, and perceived unawareness was associated with a 74 % decreased likelihood of initiating a first report.

And third, we considered whether children’s awareness was predictive of their disclosure behavior in the first investigative interview. Because of the potential importance of clear and convincing full disclosures in investigative interviews, we elected to include only those children who provided such accounts, and compare them to children who failed to disclose, leading us to omit children who gave partial disclosures in the first investigative interview (*n* = 13, all from the neglect sample). The low N of the latter group, as well, precluded our ability to include it in the analysis to test for further differences. The logistic regression predicting disclosure versus non-disclosure from age, type of maltreatment, and perceived awareness was significant, *χ*^*2*^(3, *N* = 121) = 38.35, *p* < .001, Nagelkerke’s *R*^*2*^ = 0.41 (see also Table 2). Both type of maltreatment, *b* = 1.14, *p* = .001, OR = 3.13, and perceived awareness, *b* = 1.41, *p* = .013, OR = 4.09, were significant. Children who had experienced sexual abuse were more than three times more likely to disclose in the first investigative interview than children who had experienced neglect, and children with perceived awareness were more than four times more likely to disclose in the first investigative interview than children who were perceived as unaware of the maltreatment.

## 4. Discussion

Substantiation of both neglect and child sexual abuse often relies heavily on information provided by children that can confirm the maltreatment’s occurrence and clearly identify the perpetrator. Yet, neglect and sexual abuse are discovered in very different ways, part of which may be explained by differences in awareness of the types of behaviors that are abusive, which may impact whether children disclose during an investigative interview or not. In particular, neglect was often initially discovered due to incidents involving emergency services (e.g., police, emergency medical services), and not through children’s own reports. In contrast, sexual abuse tended to be suspected due to children’s self-initiated statements, consistent with previous research on disclosures of sexual abuse (Goodman et al., 1992; London et al., 2005; London et al., 2008). These varying paths to identification were similarly reflected in the proportion of children who seemed unaware that their experiences constituted maltreatment. Children were less aware that their own experiences constituted neglect than sexual abuse, and less awareness in turn predicted decreased likelihood of a formal disclosure.

### 4.1. Initial discovery of maltreatment

Of importance, only 4 % of the neglect case file investigations in our sample were initiated due to children’s initial statements; instead, neglect was suspected through other means. However, 46 % of children did disclose details of neglect in the first investigative interview. Conversely, 58 % of sexual abuse case file investigations were initiated due to children’s initial statements, and 95 % of children disclosing details of sexual abuse in the first investigative interview. To our knowledge, this is the first direct analysis of forensic reports of neglect, and sheds light on the relatively low rates of initial reports among children who have experienced neglect compared to disclosures among children who have experienced sexual abuse.

It is possible that disclosure suspicion and substantiation bias may be partly responsible for the large gap between neglect and sexual abuse cases in terms of initial reports and disclosures in the investigative interview. As discussed in Rush et al. (2014), disclosure suspicion bias and substantiation bias refer to the fact that sexual abuse is usually suspected and ultimately substantiated based on a disclosure by the child. Applying this knowledge to our sexual abuse sample, the disclosure rates were likely so high because they were cases that had been established by social services as having enough evidence to determine that sexual abuse had occurred, and in such cases a disclosure weighs heavily.

In comparison, only roughly half of our sample of children who had experienced (substantiated) neglect disclosed in their first investigative interview, which highlights that in substantiation decisions for neglect, less weight is placed on the child’s own disclosure than is the case for substantiation decisions for sexual abuse (Leach et al., 2017; Lyon et al., 2012). Social workers may also be able to collect broader ranges of evidence for substantiating neglect than CSA, thus leading to higher rates of non-disclosure in neglect cases than sexual abuse cases because children do not have to disclose to be brought to the attention of the authorities. At the same time, the fact that children were also less aware of neglectful parenting behaviors suggests that the children were not able to recognize the problem or seek assistance by telling others about their circumstances, which we discuss next.

### 4.2. Perceived awareness and reports and disclosures

In our study, a small number of children, who seemed unaware of their own neglect, nonetheless unintentionally disclosed their circumstances in the investigative interview, displaying what Collings et al. (2005) described as “indirect disclosures” that occur when children accidentally alert another individual to a potential concern through their comments, either about abuse or the surrounding circumstances. Much more common were formal disclosures by children who were aware that they had experienced neglect or at the very least had experienced circumstances that were threatening or harmful.

Among the children who failed to initiate discovery on their own was a subset who expressed a vague disapproval in their circumstances or a parent’s behavior, but nonetheless failed to disclose in the investigative interview (e.g., saying the social worker removed the child from the home because the parent used bad words when that was not a factor that weighed in on the child’s removal). Others seemed not to know that their experiences constituted a form of neglect. Such aligns with the first stage of Finkelhor and Wolak’s (2003) two-stage model of crime reporting in which the problem must be recognized before it can be reported. In contrast, for many children who have experienced sexual abuse, unawareness did not appear to be a driving factor behind any non-disclosures.

Investigative interviewing approaches, therefore, need to address the different underlying reasons why children fail to disclose. It may be crucial to assess neglect indirectly, for instance, by asking about children’s perception of their home life, including their feelings about or experiences with their needs being met, safety, and comfort. Children’s responses may provide key insight into barriers to disclosure. Further, such questions can provide hints about awareness, and may be a valuable starting point as long as they are conducted in an open and non-suggestive manner so that they do not increase suggestibility and decrease the credibility of the child witnesses (Castelli et al., 2005). This is important to ensure that the disclosure evidence will hold up in court examinations (Castelli et al., 2005). In contrast, when sexual abuse is suspected, a high percentage of children disclose during the investigative interview (Azzopardi et al., 2019; also Hershkowitz et al., 2014), even as early as the initial substantive prompt, “Tell me why you came to talk to me” (Sternberg et al., 2001). Accordingly, questions probing awareness may not be necessary or beneficial. If children know but are unwilling to provide details, strategies specific to overcoming disclosure reluctance, such as building rapport and the use of supportive statements (e.g., Hershkowitz et al., 2014; Lavoie et al., 2019), may be more appropriate starting points.

### 4.3. Future directions, and conclusions

There are several ways in which the findings of study can be built on in the future to address existing limitations. The first is that although we did capture perceptions of awareness in the lengthy and detailed case file reports, this finding is important enough to warrant future investigations using varied methodologies. For example, one way to further build on this finding is to consider children’s awareness of the standards of their own rights for safety, care, and provision using brief visual vignette approaches to determine whether more focus and efforts are needed for educating children about their own rights. Second, subsequent research could examine understanding and disclosure patterns among children who experience other forms of maltreatment, such as physical abuse, medical neglect, or perhaps witnessing domestic violence separately. Ideally, with larger samples, not only could awareness, discovery, and disclosure be studied, but important interactions, for instance, with gender and age, could be tested to continue to advance, in meaningful ways, understanding of reporting and disclosure trends in maltreated children. Third, in our disclosure analyses, we omitted partial disclosures. The meaning of partial disclosures is an important area for follow-up. It is possible that these children were “testing the waters” by revealing some initial details, which would suggest awareness. However, it is also possible that these children were unaware of the magnitude or severity of their experiences and hence were accidentally revealing parts of their experiences without full knowledge of their harmful potential. Further, only initial reports and disclosures were considered, and we did not consider delay to disclosure or recantations, both of which will be important to evaluate in future studies on maltreatment awareness. Finally, our sample did not include children under 4 years of age. Suspicions of sexual abuse among younger children are more likely attributable to sexualized behaviors and witnessing of abuse, and less likely due to deliberate disclosure (Mian et al., 1986). Therefore, it is likely that fewer differences exist between neglect and sexual abuse cases when very young children are involved.

## 5. Conclusions

Overall, there were substantial differences between the ways in which neglect is initially discovered, versus the ways in which sexual abuse is initially discovered. Children’s perceived awareness was a significant predictor of whether a child would initiate a first report and whether a child would make an intentional disclosure in the first investigative interview. Our findings raise the question of whether children are always aware of, or have an implicit knowledge of, the social and legal norms for parenting that would increase the likelihood of intentional disclosure. They also highlight a particular consideration for children who have experienced neglect, who may have a lower awareness and consequently lower likelihood of intentionally disclosing. With the current and next wave of research on discovery, awareness, and disclosure among children who experience different forms of maltreatment, questioning approaches should continue to evolve to address the motivational and knowledge-based reasons behind why children do or do not tell when asked about their experiences.

## Figures and Tables

**Table 1:** Relations between main variables.

	Initiate 1st report	Disclose 1st interview	Awareness
*r*_*pb*_			
Age	−0.044	0.147	0.067
*χ* ^*2*^ *, p*			
Gender	8.48, 0.004	2.48, 0.115	5.28, 0.022
Type of maltreatment	66.21, < 0.001	23.68, < 0.001	29.51, < 0.001
Awareness	29.18, <0.001	18.90, < 0.001	-

**Table 2:** Unstandardized regression coefficients for logistic regression models.

	Coefficient	*S.E*.	*p*	OR	LLCI	ULCI
Model 1: perceived awareness (outcome)						
*χ*^*2*^(2, *N* = 134) = 34.06, *p* < .001, Nagelkerke’s *R*^*2*^ = 0.30						
Age	0.07	0.07	0.302	1.07	0.94	1.23
Type of maltreatment	1.08	0.20	0.000	2.97	1.99	4.43
Constant	−2.71	0.93	0.004	0.07		
Model 2: initiate 1st report (outcome)						
*χ*^*2*^(3, *N* = 133) = 88.52, *p* < .001, Nagelkerke’s *R*^*2*^ = 0.66						
Age	−0.21	0.12	0.080	0.81	0.64	1.03
Type of maltreatment	−2.06	0.38	0.000	0.13	0.06	0.27
Perceived awareness	−1.45	0.61	0.017	0.23	0.07	0.78
Constant	10.00	2.31	0.000	21,804		
Model 3: disclose 1st forensic interview (outcome)						
*χ*^*2*^(3, *N* = 121) = 38.35, *p* < .001, Nagelkerke’s *R*^*2*^ = 0.41						
Age	0.14	0.08	0.097	1.15	0.98	1.35
Type of maltreatment	1.14	0.34	0.001	3.13	1.60	6.11
Perceived awareness	1.41	0.57	0.013	4.09	1.35	12.39
Constant	−4.30	1.26	0.001	0.014		

## Data Availability

The authors do not have permission to share data.

## References

[R1] Alaggia R, Collin-Vézina D, & Lateef R (2019). Facilitators and barriers to child sexual abuse (CSA) disclosures: A research update (2000–2016). Trauma, Violence, & Abuse, 20, 260–283. 10.1177/1524838017697312 PMC6429637 29333973

[R2] Anderson GD (2016). The continuum of disclosure: Exploring factors predicting tentative disclosure of child sexual abuse allegations during forensic interviews and the implications for practice, policy, and future research. Journal of Child Sexual Abuse, 25, 382–402. 10.1080/10538712.2016.1153559 27266535

[R3] Azzopardi C, Eirich R, Rash CL, MacDonald S, & Madigan S (2019). A meta-analysis of the prevalence of child sexual abuse disclosure in forensic settings. Child Abuse & Neglect, 93, 291–304. 10.1016/j.chiabu.2018.11.020 30579645

[R4] Block SD, Oran H, Oran D, Baumrind N, & Goodman GS (2010). Abused and neglected children in court: Knowledge and attitudes. Child Abuse & Neglect, 34, 659–670. 10.1016/j.chiabu.2010.02.003 20719384

[R5] Brubacher SP, Malloy LC, Lamb ME, & Roberts KP (2013). How do interviewers and children discuss individual occurrences of alleged repeated abuse in forensic interviews? Applied Cognitive Psychology, 27, 443–450. 10.1002/acp.2920

[R6] Castelli P, Goodman GS, & Ghetti S (2005). Effects of interview style and witness age on perceptions of children’s credibility in sexual abuse cases. Journal of Applied Social Psychology, 35, 297–319.

[R7] Collings S, Griffiths S, & Kumalo M (2005). Patterns of disclosure in child sexual abuse. South African Journal of Psychology, 35, 270–285.

[R8] Dickerson K, Flynn E, Levine LL, & Quas JA (2018). Are emotions controllable? Maltreated and non-maltreated youth’s implicit beliefs about emotions and aggressive tendencies. Child Abuse and Neglect, 77, 222–231. 29407607 10.1016/j.chiabu.2018.01.010

[R9] Dickerson KL, Lavoie J, & Quas JA (2020). Do laypersons conflate poverty and neglect? Law & Human Behavior, 44, 311–326. 10.1037/lhb0000415 32673001 PMC7905956

[R10] Dubowitz H, Klockner A, Starr RH, & Black MM (1998). Community and professional definitions of child neglect. Child Maltreatment, 3, 235–243.

[R11] Finkelhor D, & Wolak J (2003). Reporting assaults against juveniles to the police: Barriers and catalysts. Journal of Interpersonal Violence, 18, 103–128. 10.1177/0886260502238730

[R12] Folman RD (1998). I was tooken. Adoption Quarterly, 2, 7–35. 10.1300/J145v02n02_02

[R13] Gonzalez D, Bethencourt MA, & McCall JD (2021). Child abuse and neglect. Treasure Island (FL): StatPearls Publishing. Available from: https://www.ncbi.nlm.nih.gov/books/NBK459146/.

[R14] Goodman GS, Taub EP, Jones DPH, England P, Port LK, Rudy L, Prado L, Myers JEB, & Melton GB (1992). Testifying in criminal court: Emotional effects on child sexual assault victims. Monographs of the Society for Research in Child Development, 57, 1–159. 10.2307/1166127 1470193

[R15] Grandgenett HM, Pittenger S, Dworkin ER, & Hansen DJ (2019). Telling a trusted adult: Factors associated with the likelihood of disclosing child sexual abuse prior to and during a forensic interview. Child Abuse & Neglect, 116, Article 104193. 10.1016/j.chiabu.2019.104193 PMC7089833 31561907

[R16] Griffin M (2008). Getting to know you? Issues of trust and mistrust in understanding community, developing partnerships and delivering policy change in children’s services. Early Child Development and Care, 180, 879–888. 10.1080/03004430802524974

[R17] Hauser C, & Astor M (2018). The Larry Nassar case: What happened and how the fallout is spreading. The New York Times. https://www.nytimes.com/2018/01/25/sports/larry-nassar-gymnastics-abuse.html.

[R18] Heger A, Ticson L, Velasquez O, & Bernier R (2002). Children referred to for possible sexual abuse: Medical findings in 2384 children. Child Abuse & Neglect, 26, 645–659. 10.1016/s0145-2134(02)00339-3 12201160

[R19] Hershkowitz I, Lamb ME, & Carmit K (2014). Allegation rates in forensic child abuse investigations: Comparing the revised and standard NICHD protocols. Psychology, Public Policy, & Law, 20, 336–344. 10.1037/a0037391

[R20] Hritz AC, Royer CE, Helm RK, Burd KA, Ojeda K, & Ceci SJ (2015). Children’s suggestibility research: Things to know before interviewing a child. Anuario de Psicologia Juridica, 25, 3–12. 10.1016/j.apj.2014.09.002

[R21] Kemshall H, & Moulden HM (2017). Communicating about child sexual abuse with the public: Learning the lessons from public awareness campaigns. Journal of Sexual Aggression, 23, 124–138. 10.1080/13552600.2016.1222004

[R22] Lavoie J, Dickerson KL, Redlich AD, & Quas JA (2019). Overcoming disclosure reluctance in youth victims of sex trafficking: New directions for research, policy, and practice. Psychology, Public Policy, and Law, 25, 225–238. 10.1037/law0000205 PMC7043240 32103880

[R23] Leach C, Powell MB, Sharman SJ, & Anglim J (2017). The relationship between children’s age and disclosures of sexual abuse during forensic interviews. Child Maltreatment, 22, 79–88. 10.1177/1077559516675723 27784813

[R24] London K, Bruck M, Ceci SJ, & Shuman DW (2005). Disclosure of child sexual abuse: What does the research tell us about the ways that children tell? Psychology, Public Policy, & Law, 11, 194–226. 10.1037/1076-8971.11.1.194

[R25] London K, Bruck M, Wright DB, & Ceci SJ (2008). Review of the contemporary literature on how children report sexual abuse to others: Findings, methodological issues, and implications for forensic interviewers. Memory, 16, 29–47. 10.1080/09658210701725732 18158687

[R26] Lyon TD, Ahern EC, & Scurich N (2012). Interviewing children versus tossing coins: Accurately assessing the diagnosticity of children’s disclosures of abuse. Journal of Child Sexual Abuse, 21, 19–44. 10.1080/10538712.2012.642468 22339423 PMC3982784

[R27] Malloy LC, Katz C, Lamb ME, & Mugno AP (2015). Children’s requests for clarification in investigative interviews about suspected sexual abuse. Applied Cognitive Psychology, 29, 323–333. 10.1002/acp.3101

[R28] Malloy LC, Lyon TD, & Quas JA (2007). Filial dependency and recantation of child sexual abuse allegations. Journal of the American Academy of Child and Adolescent Psychiatry, 46, 162–170. 10.1097/01.chi.0000246067.77953.f7 17242619

[R29] Malloy LC, Mugno AP, Rivard JR, Lyon TD, & Quas JA (2016). Familial influences on recantation in substantiated child sexual abuse cases. Child Maltreatment, 21, 256–261. 10.1177/1077559516650936 27234520 PMC6353559

[R30] McDaniel M (2006). In the eye of the beholder: The role of reporters in bringing families to the attention of child protective services. Children and Youth Services Review, 28, 306–324. 10.1016/j.childyouth.2005.04.010

[R31] McElvaney R, Moore K, O’Reilly K, Turner R, Walsh B, & Guerin S (2020). Child sexual abuse disclosures: Does age make a difference? Child Abuse & Neglect, 99, Article 104121. 10.1016/j.chiabu.2019.104121 31838224

[R32] Meysen T, & Kelly L (2017). Child protection systems between professional cooperation and trustful relationships: A comparison of professional practical and ethical dilemmas in England/Wales, Germany, Portugal, and Slovenia. Child & Family Social Work, 23, 222–229. 10.1111/cfs.12403

[R33] Mian M, Wehrspann W, Klajner-Diamond H, Lebaron D, & Winder C (1986). Review of 125 children 6 years of age and under who were sexually abused. Child Abuse & Neglect, 10, 223–229. 3708426 10.1016/0145-2134(86)90083-9

[R34] Okur P, van der Knaap LM, & Bogaerts S (2020). A quantitative study on gender differences in disclosing child sexual abuse and reasons for nondisclosure. Journal of Interpersonal Violence, 35, 5255–5275. 10.1177/0886260517720732 29294841

[R35] Portwood SG (1998). The impact of individuals’ characteristics and experiences on their definitions of child maltreatment. Child Abuse & Neglect, 22, 437–452. 10.1016/S0145-2134(98)00008-8 9631254

[R36] Priebe G, & Svedin CG (2008). Child sexual abuse is largely hidden from the adult society. An epidemiological study of adolescents’ disclosures. Child Abuse & Neglect, 32, 1095–1108. 10.1016/j.chiabu.2008.04.001 19038448

[R37] Quas JA, Malloy LC, Melinder A, Goodman GS, D’Mello M, & Schaaf J (2007). Developmental differences in the effects of repeated interviews and interviewer bias on young children’s event memory and false reports. Developmental Psychology, 43, 823–837. 10.1037/0012-1649.43.4.823 17605517 PMC2913698

[R38] Quas JA, Wallin AR, Horwitz B, Davis E, & Lyon TD (2009). Maltreated children’s understanding of and emotional reactions to dependency court involvement. Behavioral Sciences & the Law, 27, 97–117. 10.1002/bsl.836 19156680 PMC2856467

[R39] Rose SJ, & Selwyn J (2000). Child neglect: An English perspective. International Social Work, 43, 179–192. 10.1177/002087280004300204

[R40] Rush EB, Lyon TD, Ahern EC, & Quas JA (2014). Disclosure suspicion bias and abuse disclosure: Comparisons between sexual and physical abuse. Child Maltreatment, 19, 113–118. 10.1177/1077559514538114 24899582 PMC4256129

[R41] Rush EB, Stolzenberg SN, Quas JA, & Lyon TD (2017). The effects of the putative confession and parent suggestion on children’s disclosure of a minor transgression. Legal and Criminological Psychology, 22, 60–73. 10.1111/lcrp.12086 28286409 PMC5342253

[R42] Saywitz KJ, Lyon TD, & Goodman GS (2018). When interviewing children: A review and update. In Klika JB, & Conte JR (Eds.), APSAC handbook on child maltreatment (4th ed., pp. 310–329). Newbury Park, CA: Sage.

[R43] Sternberg KJ, Lamb ME, Orbach Y, Esplin PW, & Mitchell S (2001). Use of a structured investigative protocol enhances young children’s responses to free-recall prompts in the course of forensic interviews. Journal of Applied Psychology, 86, 997–1005. 10.1037/0021-9010.86.5.997 11596815

[R44] Administration on Children, Y.a. F. U.S. Department of Health &, Human Services, Administration for Children and Families, Children’s Bureau. (2019). Child maltreatment 2017. Available from.

[R45] Wandrey L, Lyon TD, Quas JA, & Friedman WJ (2012). Maltreated children’s ability to estimate temporal location and numerosity of placement changes and court visits. Psychology, Public Policy, and Law, 18, 79–104. 10.1037/a0024812 PMC3280883 22347789

